# Complex Management of a Persistent Gastropleural Fistula With Necrotic Left Lower Lobe of the Lung Following Bariatric Surgery: A Case Report

**DOI:** 10.7759/cureus.108602

**Published:** 2026-05-10

**Authors:** Yamam Al-Shamary, Hemangi Chavan, Sofina Begum

**Affiliations:** 1 Department of Thoracic Surgery, Royal Brompton Hospital, London, GBR

**Keywords:** bariatric surgery complication, gastropleural fistula, left lower lobe necrosis, postoperative fistula, pulmonary necrosis

## Abstract

Gastropleural fistula is a rare but serious complication characterised by an abnormal communication between the stomach and the pleural cavity. It poses substantial diagnostic and therapeutic challenges and often requires coordinated multidisciplinary intervention. We report the case of a 52-year-old woman with a complex surgical history, including multiple bariatric procedures, who developed a persistent gastropleural fistula complicated by necrosis of the left lower lung lobe. Multiple endoscopic interventions, including Ovesco clip placement and fibrin glue injection, were unsuccessful in achieving fistula closure. Clinically, she presented with a chronic productive cough yielding greenish sputum, along with coarse crepitations localised to the left lower lung zone. Imaging demonstrated a left-sided pleural effusion, prompting further evaluation. Contrast-enhanced CT of the chest and abdomen revealed a patent fistulous tract communicating with the proximal gastric body and extensive left lower lobe necrosis. Surgical management included left lower lobectomy, circumferential dissection, and identification of the gastropleural fistula, transposition of the fistulous tract into the abdominal cavity, and primary diaphragmatic repair to prevent continued leakage into the thoracic cavity. Following surgery, the patient was transferred to a specialised upper gastrointestinal centre for ongoing care and definitive fistula management. This case highlights the profound impact gastropleural fistulas can have on pulmonary structures and underscores the complexity of their management. It emphasises the necessity of a multidisciplinary approach integrating thoracic and upper gastrointestinal surgical expertise to optimise outcomes.

## Introduction

A gastropleural fistula (GPF) is a rare pathological communication between the stomach and the pleural cavity, first described by Markowitz and Herter in 1960 [1]. It remains an uncommon but challenging condition that often requires a multidisciplinary approach. GPF most frequently arises as a complication of upper gastrointestinal surgery, particularly bariatric procedures such as sleeve gastrectomy or biliopancreatic diversion [2,3]. Less commonly, it may result from malignancy, trauma, perforated hiatal hernia, lung surgery or post-chemotherapy or radiotherapy [1,4-6].

The incidence of GPF following bariatric surgery is extremely low, reported at 0.2-0.37% of postoperative patients [7]. A French multicentre study including 24 patients with gastropleural or gastrobronchial fistulas reported a healing success rate of approximately 90% after first-line and secondary interventions, though morbidity was high (42%) and resolution often required several months [8].

This case is notable for the rare and severe complication of a persistent GPF with extensive pulmonary involvement, including left lower lobe necrosis. Unlike most cases, where lung involvement is minimal, this patient required thoracic intervention to control pulmonary infection and necrosis before definitive gastrointestinal repair. This report provides insights into the multidisciplinary management of complex cases in which both the gastrointestinal tract and pulmonary parenchyma are severely affected.

Clinical presentation varies with fistula size and chronicity. Some patients present acutely postoperatively, while others develop subtle, nonspecific symptoms such as persistent cough, epigastric or chest pain, or shortness of breath [3,9]. In patients with unresolved or recurrent pneumonia after gastric surgery, GPF should be considered [9]. The presence of food particles or bile in chest drainage fluid is a highly suggestive diagnostic clue.

Radiological evaluation typically begins with a chest X-ray, which may show pneumonia, pleural effusion, pneumothorax, or hydropneumothorax [9]. Contrast-enhanced CT is particularly useful for localising the fistula and assessing pulmonary and pleural involvement. Conservative management, including nil-by-mouth status, total parenteral nutrition, or jejunal feeding, may be attempted in minimally symptomatic patients [9]. Persistent or complicated fistulas, however, often require definitive surgical intervention [10].

We present a case of a persistent gastropleural fistula causing left pleural effusion and left lower lobe necrosis. The patient underwent left lower lobectomy and isolation of the fistula from the thoracic cavity, followed by transfer to a specialised upper gastrointestinal centre for ongoing management and definitive repair.

## Case presentation

History

A 52-year-old woman transferred to our hospital for the surgical management of a gastropleural fistula and necrotic left lower lobe following a complex bariatric surgical history (Table 1).

**Table 1:** Patient's bariatric surgical history

Year	Procedure / Intervention	Outcome / Complication
2006	Gastric band insertion	Later removed due to intolerance
2016	Roux-en-Y gastric bypass	Uneventful
Mar 2024	Revision to Single Anastomosis Duodeno–Ileal Switch (SADIS)	Complicated by upper OG leak and gastropleural fistula
Mar 2024	Laparoscopic drainage and washouts	Temporary control of infection, fistula persisted
Aug 2024	Endoscopic Ovesco clip placement	Failed to close fistula
Nov 2024	Lipiodol/histoacryl glue injection	Partial response, fistula persisted
Jan 2025	Antibiotic sterilisation and repeated glue application	No definitive closure

Our patient was symptomatically stable, presented mainly with a persistent productive cough producing greenish sputum, but without fever, chest pain, dyspnea, or epigastric pain. Nutritional support was ongoing via nasojejunal feeding and total parenteral nutrition (TPN).

Examination

She was comfortable and showed no signs of respiratory distress. A nasojejunal tube was in situ on free drainage with TPN running. Vital signs were within normal limits, and chest examination showed bilateral air entry with coarse crepitations in the left lower zone. The abdomen was soft, non-distended, and non-tender with bowel sounds present.

Investigations

**Figure 1:**
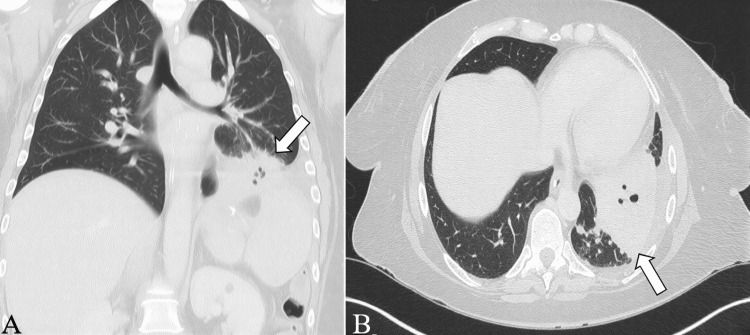
Contrast-enhanced thorax and abdominal CT, arterial phase. (A) Coronal section - Small collection with air foci within noted at the site of the proximal previous sleeve staple line, this collection is continuing into left pleural space collection through the left hemidiaphragm. (B) Axial section - Necrotic left lower lobe

Laboratory investigations on admission are summarised in Table 2. Additionally, a contrast-enhanced thorax and abdominal CT was performed, and confirmed appearances were in keeping with a patent gastropleural fistula from the proximal aspect of the gastric sleeve suture line in the body of the stomach extending to a subphrenic collection which communicated with a left basal pleuropulmonary cavity. In addition, there was a large, rounded area of consolidation within the left lower lobe containing low-attenuation regions consistent with necrosis or cavitation. A small left-sided pleural effusion was also noted (Figure 1).

**Table 2:** Laboratory investigations on admission

Parameters	Patient Values	Reference Range
Hemoglobin	111 g/L	115–151 g/L
White Blood Cell Count	7.3 × 10⁹/L	4.4–10.1 × 10⁹/L
Platelet Count	253 × 10⁹/L	136–343 × 10⁹/L
C-Reactive Protein	46 mg/L	<5 mg/L
Creatinine	36 µmol/L	60–120 µmol/L
Alkaline Phosphatase	131 U/L	30–130 U/L
Total Bilirubin	4 µmol/L	<21 µmol/L
Alanine Transaminase	23 U/L	0–35 U/L

Management

A two-stage surgical approach was planned in collaboration with the upper gastrointestinal team. The first stage aimed to control thoracic complications, including ongoing infection, pleural contamination, and necrotic lung tissue, minimising operative risk. The second stage involved transfer to a specialised centre for definitive gastrointestinal management.

Preoperative Care

Broad-spectrum intravenous antibiotics covering gram-positive, gram-negative, and anaerobic organisms were administered. General anaesthesia with single-lung ventilation was used to optimise exposure and protect the contralateral lung. Intraoperative monitoring included arterial pressure, central venous access, urine output, and serial arterial blood gases.

Surgical Procedure

**Figure 2:**
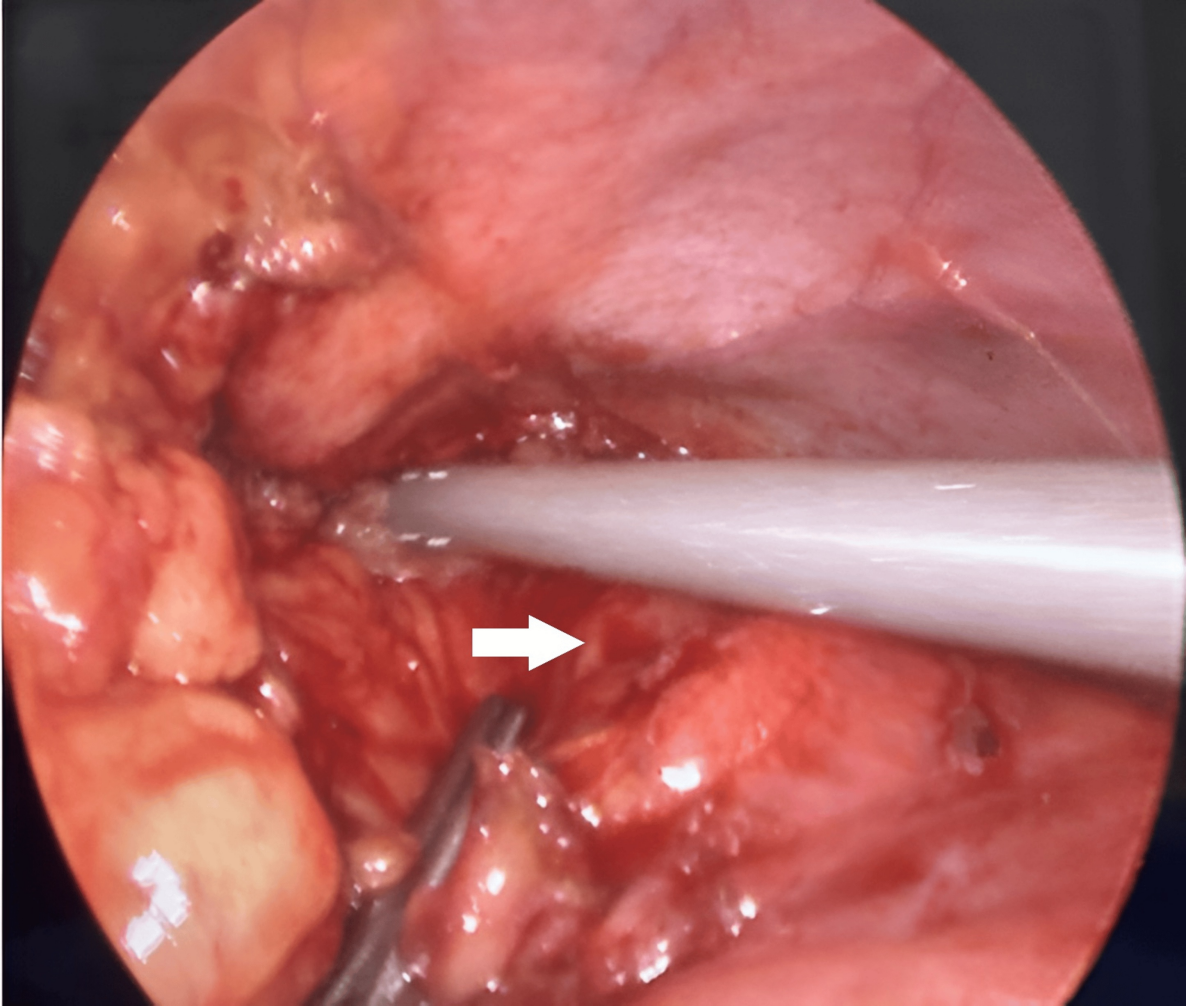
The fistula was successfully excluded from the thoracic cavity and contained within the left subphrenic region of the abdominal cavity.

Bronchoscopy and esophagogastroduodenoscopy assessed airway patency and fistula anatomy. A left thoracotomy revealed purulent pleural fluid, dense adhesions, and necrosis of the left lower lobe. The pleural cavity was irrigated, and decortication was performed to free the lung. Circumferential dissection isolated the fistula, which was then transposed into the abdominal cavity. The diaphragm was repaired to prevent further thoracic involvement. A left lower lobectomy was completed, and laparoscopic confirmation confirmed complete exclusion of the fistula within the left subphrenic space (Figure 2).

Postoperatively, the patient recovered without complications and was transferred on post-operative day 8 to a specialised upper gastrointestinal centre for definitive management. Histopathology confirmed acute and chronic bronchopneumonia, consistent with chronic fistulous contamination, with no malignancy.

## Discussion

GPFs can occur as an early or late complication of bariatric surgery. It has been reported as early as seven days after surgery [8] and as late as 13 years post-procedure [11]. Our patient developed GPF two weeks after undergoing a single anastomosis duodeno-ileal switch surgery. GPFs are notoriously difficult to treat. The exact mechanism of formation after bariatric procedures is not fully understood, but is thought to involve a postoperative gastric leak at the staple line leading to subphrenic infection and subsequent erosion through the diaphragm into the pleural cavity [3,11]. Persistent inflammation may result in the development of a mature fistulous tract. Infected collections and ongoing leakage of gastric contents can contribute to pulmonary complications, including infection and necrosis. In addition, pressure gradients are believed to play a role, as evidenced by the need to control airway pressures to reduce bronchopleural fistula output and promote healing; by analogy, negative intrathoracic pressure may contribute to persistence of gastropleural fistulas by promoting the movement of gastric contents into the pleural cavity [12]. Chronic leakage leads to inflammation, fibrosis, and an epithelialized tract, making spontaneous closure or endoscopic repair unlikely. Repeated interventions may exacerbate tissue damage and alter anatomy, complicating definitive repair.

In this case, the pulmonary necrosis is most likely secondary to chronic contamination of the pleural cavity by gastric contents due to the persistent fistula, leading to ongoing infection, inflammation, and ultimately necrosis of the left lower lobe. Contrast-enhanced CT demonstrated a clear fistulous communication, supporting this pathophysiological mechanism. Similar cases in the literature describe complex post-bariatric GPFs requiring en bloc resection or decortication following failure of conservative or endoscopic therapies [11,13,14]. The persistence of the fistula in this patient despite multiple interventions is consistent with the most refractory cases reported.

Prognosis and complications

Timely recognition, effective source control, and ongoing management are critical. Potential complications include recurrent pleural infection, empyema, further lung destruction, respiratory failure, nutritional compromise, and fistula recurrence.

Surgical strategy

A staged surgical approach was employed, with initial thoracic source control comprising decortication, lobectomy, and exclusion of the fistula, followed by delayed definitive gastrointestinal repair. This sequence enabled physiological stabilisation, resection of necrotic lung tissue, and control of ongoing intrathoracic contamination prior to reconstruction. Although infrequently reported, this approach may provide a useful framework for managing similarly complex cases.

Clinical recommendations

A high index of suspicion for GPF should be maintained in bariatric patients presenting with recurrent or atypical pulmonary symptoms. Early contrast-enhanced CT is recommended to delineate the fistula tract and assess the extent of pulmonary involvement. Prompt multidisciplinary evaluation, involving thoracic surgery, upper gastrointestinal surgery, radiology, and nutritional support, is essential to guide decision-making and plan staged interventions where appropriate.

There remains a need for standardised management algorithms, comparative outcome data between endoscopic and staged surgical approaches, and long-term registry data to better define risk factors, inform patient selection, and optimise the timing of definitive repair.

## Conclusions

Early recognition of gastropleural fistulas is essential to prevent severe pulmonary complications. In complex presentations, a staged surgical strategy beginning with thoracic source control and followed by definitive gastrointestinal repair may provide effective management and improve outcomes. Further work, including the development of standardised management protocols, comparative outcome studies, and long-term registries, is needed to better inform care in these challenging cases.
